# Structural basis of Naa20 activity towards a canonical NatB substrate

**DOI:** 10.1038/s42003-020-01546-4

**Published:** 2021-01-04

**Authors:** Dominik Layer, Jürgen Kopp, Miriam Fontanillo, Maja Köhn, Karine Lapouge, Irmgard Sinning

**Affiliations:** 1 Biochemiezentrum der Universität Heidelberg (BZH), Heidelberg University, INF 328, 69120 Heidelberg, Germany; 2 European Molecular Biology Laboratory, Genome Biology Unit, Meyerhofstrasse 1, 69117 Heidelberg, Germany; 3 Signalling Research Centres BIOSS and CIBSS, and Faculty of Biology, University of Freiburg, Schänzlestr. 18, 79104 Freiburg, Germany

**Keywords:** X-ray crystallography, Enzyme mechanisms

## Abstract

N-terminal acetylation is one of the most common protein modifications in eukaryotes and is carried out by N-terminal acetyltransferases (NATs). It plays important roles in protein homeostasis, localization, and interactions and is linked to various human diseases. NatB, one of the major co-translationally active NATs, is composed of the catalytic subunit Naa20 and the auxiliary subunit Naa25, and acetylates about 20% of the proteome. Here we show that NatB substrate specificity and catalytic mechanism are conserved among eukaryotes, and that Naa20 alone is able to acetylate NatB substrates in vitro. We show that Naa25 increases the Naa20 substrate affinity, and identify residues important for peptide binding and acetylation activity. We present the first Naa20 crystal structure in complex with the competitive inhibitor CoA-Ac-MDEL. Our findings demonstrate how Naa20 binds its substrates in the absence of Naa25 and support prospective endeavors to derive specific NAT inhibitors for drug development.

## Abstract (web summary)

Layer et al. present a crystal structure of Naa20, the catalytic subunit of an N-terminal acetyltransferase NatB, in complex with its competitive inhibitor CoA-Ac-MDEL. They find that Naa20 alone can acetylate NatB in vitro while Naa25, the auxiliary subunit of Naa20, increases the substrate affinity of Naa20. This study provides insights into the development of NAT inhibitors.

## Introduction

Nα-acetylation is the most common protein modification in eukaryotes. Approximately 60% of all soluble proteins in yeast, more than 70% in plants, and 80–90% in humans are N-terminally acetylated^1–3^. This modification is involved in many cellular processes affecting the stability, folding and degradation of proteins, protein interactions, subcellular localization and it is linked with several human diseases like cancer, Parkinson or Huntington disease^4–6^. During N-terminal acetylation, an acetyl group is transferred from acetyl coenzyme A (AcCoA) to the α-amino group of a polypeptide. This modification is mostly carried out co-translationally^7^ by Nα-acetyltransferase complexes (NATs), which differ in subunit composition and substrate specificity, but comprise at least one catalytic subunit^1,8^. Eight different eukaryotic NATs have been identified so far (NatA to NatH)^9^. The NatB-complex, one of the major NATs, is composed of two subunits, the catalytic subunit Naa20 and the auxiliary subunit Naa25 (formerly known as Nat3 and Mdm20, respectively)^10^. Both subunits are conserved within eukaryotic model organisms and the NatB complex is found associated with the ribosome^11–13^. Deletion mutants of *NAA25* and *NAA20* in *Saccharomyces cerevisiae* show a slow growth phenotype, are unable to form actin cables, have a defect in vacuolar and mitochondrial inheritance and are sensitive to DNA-damage causing agents^3,14–16^. Recently, ScNaa20 dependent acetylation was suggested to have a protective function in regard to protein degradation and a role in protein synthesis^17–19^. An involvement of the NatB complex in vacuolar protein sorting and cell wall maintenance, as well as in influencing the shutoff activity of influenza A virus was also suggested^20,21^. Additionally, NatB is involved in the regulation of plant development, abiotic stress response and is linked to the microRNA pathway^22,23^ and in human cell proliferation, cell survival and liver cancer progression^13,24–26^. Noteworthy, as NatB subunits exhibit partly divergent phenotypes, it was speculated that both subunits may have functions independent from each other^24,27^, and Naa25 was found to not always coexpress with Naa20 in mouse neurons^28^. Importantly, Naa25 was shown to be essential for the activity of Naa20^15,16,24^ and localizes in the cytoplasm, while Naa20 is found in both, the nucleus^13^ and cytoplasm independent of Naa25^11,13^.

NatB acts on substrates presenting N-termini with the initial methionine, which is retained and directly acetylated, followed by an acidic residue (MD-, ME-, MN- or MQ-)^3,13,16,23,29,30^. Recently, crystal structures of *Candida albicans* NatB in complex with the bisubstrate inhibitor CoA-Ac-MDSEVA and in the free state were reported^30^. They show that Naa20 adopts the canonical Gcn5-related N-acetyltransferase (GNAT) fold and accommodates a peptide at its substrate binding pocket. Naa25 forms a horse-shoe-like structure holding Naa20, and overall the *Ca*NatB structures resemble the human NatB cryo-EM structure visualizing the high evolutionary conservation^31^. However, while for the isolated catalytic subunits Naa10 and Naa50 crystal structures were reported^32,33^, structural information on Naa20 were not available so far.

In order to dissect the molecular mechanism of NatB and its use as a potential therapeutic target, we studied *Chaetomium thermophilum* NatB complex and its individual subunits. Our results show that *Ct*Naa20 is active towards a canonical NatB substrate without *Ct*Naa25 in vitro, however less efficient than in NatB. We designed, synthesized and characterized the NatB inhibitor CoA-Ac-MDEL and solved the crystal structure of the catalytic subunit Naa20 in complex with this inhibitor. The structure reveals the basis of *Ct*Naa20 substrate binding and activity towards the MDEL peptide.

## Results

### *Ct*Naa20 binds with high affinity to *Ct*Naa25

**Fig. 1:**
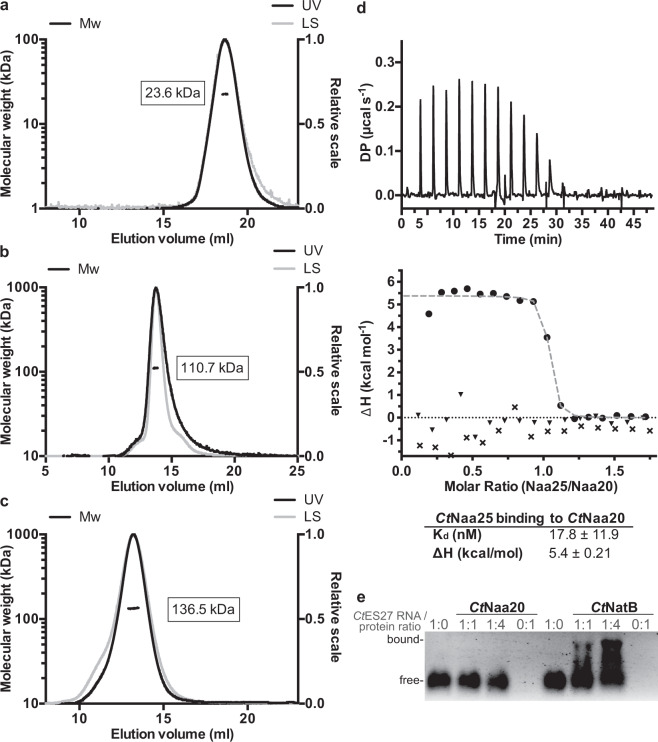
Characterization of *Ct*NatB and its subunits. **a–c** SEC-MALS analysis of *Ct*Naa20 (**a**), *Ct*Naa25 (**b**), and *Ct*NatB (**c**). The experimentally determined molecular weight of *Ct*NatB, *Ct*Naa25, and *Ct*Naa20 is 136.5 kDa (theoretical molecular is 136.1 kDa), 110.7 kDa (theoretical M_w_ is 115.1 kDa), and 23.6 kDa (theoretical M_w_ is 23.1 kDa), respectively. The UV-signals (black) of the corresponding SEC chromatograms are shown together with the light scattering signals (gray) and the mass distributions (black dots). **d** Isothermal titration calorimetry measurement of *Ct*NatB complex formation. *Ct*Naa20 was titrated by *Ct*Naa25. The signal of one representative measurement is given in differential points (DP) and the dissociation constant K_d_ and binding enthalpy are given in the table. The heats of dilution of the buffer to *Ct*Naa20 (triangle) and *Ct*Naa25 to buffer (cross) control runs are represented. Measurements were performed in triplicate and the values represent the means and standard deviations. **e** Electrophoretic mobility shift assay with the tip of *Ct*ES27 (expansion segment 27) RNA, *Ct*Naa20, and *Ct*NatB. *Ct*Naa20 and *Ct*NatB were mixed in different ratios with the RNA and free or bound RNA is indicated.

To functionally and structurally characterize the NatB complex and the subunits Naa20 and Naa25, we used the conserved orthologous proteins of the thermophilic model organism *Chaetomium thermophilum* (*Ct*) to benefit from their often superior properties in biochemical and structural studies^34,35^. A BLAST search using the *Candida albicans* (*Ca*) NAA20 and NAA25 sequences as query revealed two candidates with 42% and 20% amino acids sequence identity, respectively (Supplementary Fig. 1). Next, full length *Ct*Naa25, *Ct*Naa20 and a truncated *Ct*Naa20_1-166_ variant were cloned and expressed in *E. coli*. The *Ct*Naa20_1-166_ was based on secondary structure prediction and comprises the predicted GNAT-fold. The auxiliary subunit *Ct*Naa25 and the catalytic subunit *Ct*Naa20 were individually expressed and purified to homogeneity (Fig. 1a, b). Co-expression of *Ct*Naa25 and *Ct*Naa20 led to the formation of the NatB complex, which could also be purified to homogeneity (Fig. 1c). In order to characterize the NatB complex, size exclusion chromatography coupled to multi angle light scattering (SEC-MALS) was carried out. The individual subunits *Ct*Naa25 and *Ct*Naa20 and NatB eluted as single symmetric peaks and analyses of the molecular mass showed that *Ct*Naa20 and CtNaa25 are monomers in solution (Fig. 1a, b). A molecular mass of 136.5 kDa determined for NatB confirms a 1:1 stoichiometry of its subunits (Fig. 1c). To further characterize NatB complex formation, we performed isothermal titration calorimetry experiments. Titration of *Ct*Naa25 into *Ct*Naa20 was endothermic (Δ*H* = 5.4 kcal/mol) and resulted in the formation of a stable complex with a dissociation constant K_d_ of 17.8 ± 11.9 nM and a molar ratio of one (Fig. 1d).

As NatB acts co-translationally at the ribosome, we wanted to address ribosome binding of *Ct*Na20 and *Ct*NaB. Electrophoretic mobility shift assays (EMSA) were performed to analyze *Ct*Naa20 or *Ct*NatB binding to rRNA as indicator for putative ribosome interaction. *Ct*Naa20 and *Ct*NatB were incubated with a *C. thermophilum* expansion segment 27 RNA (*Ct*ES27) fragment. ES27 was shown to be involved in the ribosome binding of the ribosome-associated factors NatA, Arx1, and Ebp1^36–38^. A shift of the RNA band was observed upon addition of *Ct*NatB but not for *Ct*Naa20, indicating that *Ct*NatB but not *Ct*Naa20 alone can bind to *Ct*ES27 (Fig. 1e). To analyze the specificity of the interaction, hammerhead ribozyme RNA was used as control (Supplementary Fig. 2). No RNA binding for *Ct*Naa20 could be detected, while *Ct*NatB binding to hammerhead RNA was observed, showing that *Ct*NatB binds nonspecifically to RNA. This suggests that Naa20 does not associate with the ribosome on its own, but only when in complex with Naa25.

### *Ct*NatB acetylates specifically the MDEL peptide

**Fig. 2:**
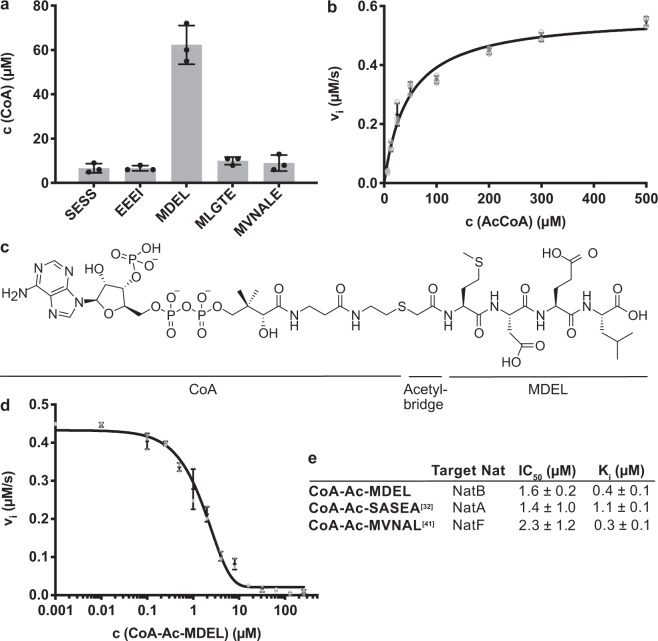
*Ct*NatB acetylation activity and inhibition. **a** Substrate specificity of *Ct*NatB tested with five different peptides. SESS, EEEI, MDEL, MLGTE and MVNALE were previously identified as NatA, Naa10/Naa80, NatB and NatC/E/F substrates. **b** Michaelis–Menten curve of the *Ct*NatB mediated acetylation of MDEL. **c** Structure of the bisubstrate analog CoA-Ac-MDEL. **d** Dose-response curve of the NatB mediated acetylation, inhibited by CoA-Ac-MDEL. **e** Inhibitor characteristics of CoA-Ac-MDEL compared to other bisubstrate analogs^32,41^. All reactions were performed in triplicates and error bars represent the standard deviation.

We then investigated *Ct*NatB substrate specificity and enzymatic activity by in vitro acetylation assays. Canonical NatA (SESS)^1^, Naa80/Naa10 (EEEI)^39,40^, NatB (MDEL)^13^, and NatC/E/F (MVNALE and MLGTE)^3^ substrates were tested. *Ct*NatB acetylates only the MDEL peptide, highlighting that NatB specificity is conserved (Fig. 2a). The MDEL peptide was then used to determine the NatB enzymatic parameters. *Ct*NatB showed a Michaelis constant (K_m_) of 45.6 ± 4.8 μM for AcCoA and a turnover number (k_cat_) of 68.8 ± 2.0 min^−1^ (Fig. 2b). These values are in good agreement with the values observed for *Ca*NatB and *At*NatB (Supplementary Fig. 3a)^23,30^. Based on these results, we designed and synthesized a bisubstrate analog, CoA-Ac-MDEL (Fig. 2c and Supplementary Fig. 3b). This bisubstrate is a potent competitive NatB inhibitor with a half-maximum inhibitor concentration (IC_50_) of 1.56 ± 0.24 μM and an inhibitor constant K_i_ of 0.41 ± 0.14 μM (Fig. 2d and Supplementary Fig. 3c). The potency of CoA-Ac-MDEL in inhibiting *Ct*NatB is in the same range as CoA-Ac-MVNAL inhibiting NatF and CoA-SASEA inhibiting NatA (Fig. 2e)^32,41^. Taken together, our results show that NatB substrate specificity is evolutionarily conserved, and indicate that all NATs bind their specific inhibitors with similar affinities.

### *Ct*Naa20 is active and specifically acetylates the NatB substrate MDEL

**Fig. 3:**
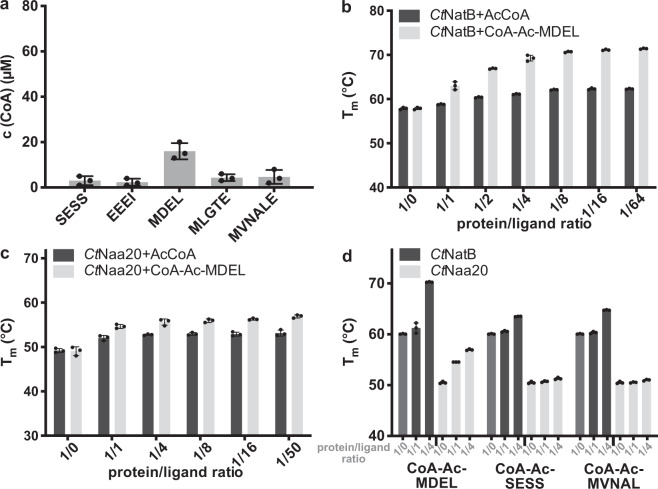
*Ct*Naa20 acetylation activity and *Ct*Naa20/*Ct*NatB-ligand interaction. **a** Substrate specificity of *Ct*Naa20 tested with five different peptides. **b** Melting temperatures of *Ct*NatB in the presence of varying concentrations of AcCoA and CoA-Ac-MDEL. **c** Melting temperatures of *Ct*Naa20 in the presence of varying concentrations of AcCoA and CoA-Ac-MDEL. **d** Melting temperatures of *Ct*NatB and *Ct*Naa20 in the presence of varying concentrations of CoA-Ac-MDEL, CoA-Ac-SESS, or CoA-Ac-MVNAL. All measurements were performed in triplicates and error bars represent the standard deviation.

To further characterize *Ct*NatB, we investigated the activity of the catalytic subunit *Ct*Naa20 alone. In contrast to previous reports on NatB, *Ct*Naa20 shows a clearly detectable and specific activity towards the MDEL peptide (Fig. 3a) with a K_m_ value for AcCoA of 12.0 ± 1.0 μM and a k_cat_ of 9.0 ± 0.2 min^−1^ (Supplementary Fig. 4a). These data show that the catalytic subunit alone is active, but less efficient than in complex with *Ct*Naa25. The bisubstrate analog CoA-Ac-MDEL is also a potent inhibitor of *Ct*Naa20, with an IC_50_ of 6.5 ± 2.5 μM (Supplementary Fig. 4b). Compared to NatB the higher IC_50_ indicates a lower affinity of *Ct*Naa20 to this inhibitor. The difference between the enzymatic activities of *Ct*Naa20 alone and as part of NatB might be explained by different affinities for the MDEL substrate. To test this, we performed kinetic experiments with constant AcCoA, but varying MDEL concentrations. *Ct*NatB shows a K_m_ of 232 ± 28 μM for MDEL, which is significantly lower than the one of *Ct*Naa20 alone (4.4 ± 0.9 mM), while the k_cat_ is in a similar range (Supplementary Fig. 4c, d). These data suggest that *Ct*Naa25 increases the affinity of *Ct*Naa20 for NatB substrates.

To further investigate the *Ct*NatB and *Ct*Naa20 ligand interactions, we performed nanoDSF based binding assays using CoA-Ac-MDEL and AcCoA. Binding of both ligands to *Ct*NatB and *Ct*Naa20 is indicated by protein stabilization (Fig. 3b, c). When AcCoA is added to *Ct*NatB, a mild stabilizing effect was observed by an increase of the unfolding transition temperature from 58 °C to 62 °C (using a 1/64 ratio of protein/ligand). Upon addition of the bisubstrate analog, *Ct*NatB melting temperatures increased drastically from 58 °C to 71 °C. This illustrates a major contribution of the NatB specific peptide to the stabilization of the protein, compared to AcCoA alone (Fig. 3b). Noteworthy, the bisubstrate has a stronger effect on *Ct*Naa20 melting temperature increase than AcCoA. These data confirm that *Ct*Naa20 alone is able to bind MDEL and support the observation that it can acetylate MDEL without *Ct*Naa25 (Fig. 3c). As a control we used two similar bisubstrate analogs with different peptide moieties in the nanoDSF assay (Fig. 3d). CoA-Ac-SESS and CoA-Ac-MVNAL were described as NatA and NatF inhibitors, respectively^41–43^. As they contain a CoA moiety, they exhibit a mild stabilizing effect on *Ct*NatB, which is significantly lower than the effect of CoA-Ac-MDEL. The difference in stabilization highlights that MDEL binds to *Ct*Naa20, but not the other peptides. Noteworthy, when only the MDEL peptide was added in a saturating amount to *Ct*NatB or *Ct*Naa20, no significant stabilizing effect was detected (Supplementary Fig. 4e). This is in accordance with the mechanism reported for Naa50, where AcCoA needs to bind before a substrate can bind^44^. Taken together, our data clearly show that *Ct*Naa20 is active towards a canonical NatB substrate without *Ct*Naa25. However, *Ct*Naa25 increases the *Ct*Naa20 affinity for this substrate, allowing for a more efficient acetylation.

### *Ct*Naa20 crystal structure in complex with CoA-Ac-MDEL

**Fig. 4:**
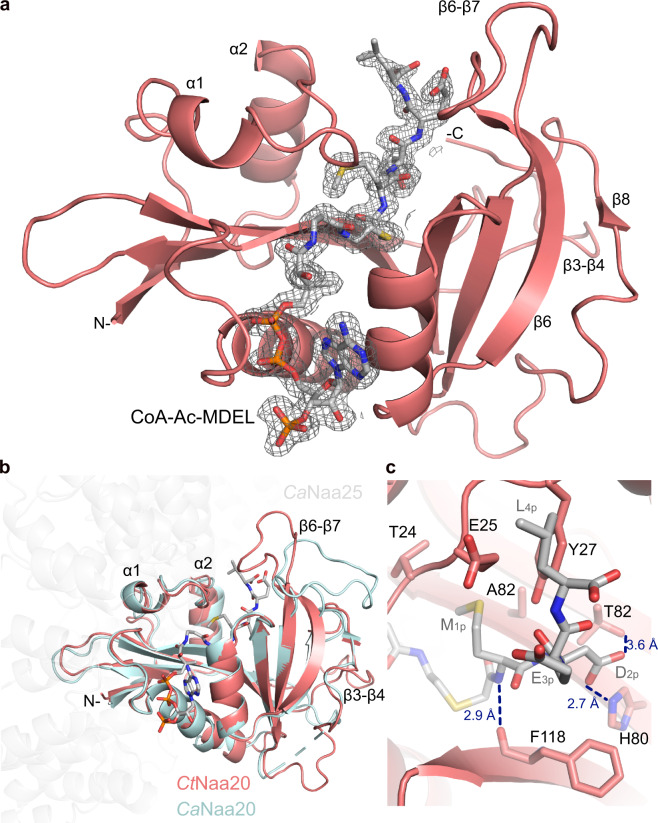
*Ct*Naa20 crystal structure. **a** Overall structure of *Ct*Naa20 with CoA-Ac-MDEL. *Ct*Naa20 is shown in red as a cartoon and CoA-Ac-MDEL as gray sticks with nitrogen, oxygen, phosphorus, and sulfur shown in blue, red, orange, and yellow, respectively. The 2mF_obs_-DF_cal_ electron density around the bisubstrate is shown at a contour level of 1σ (gray mesh). **b** The *Ct*Naa20 structure is superimposed to *Ca*Naa20 as part of CaNatB (pdb: 5k18^30^). Parts of the *Ca*Naa25 structure are shown transparent to clarify the orientation. **c** Hydrogen bonds between *Ct*Naa20 and CoA-Ac-MDEL are visualized with blue dotted lines and the corresponding atom distances.

So far, structural information on Naa20 in the absence of the adaptor subunit Naa25 has not been available. In order to characterize Naa20 on an atomic level, we crystallized *Ct*Naa20 in complex with the bisubstrate analog CoA-Ac-MDEL. The structure was solved in space group P 2_1_ with two molecules per asymmetric unit. The initial phases were obtained by molecular replacement with the *Ca*Naa20 structure part of the *Ca*NatB complex (pdb: 5k18)^30^. The structure could be built at 1.57 Å resolution, revealing the expected GNAT-fold (Table 1; Fig. 4a). The high quality of the electron density map allowed building residues 2–190 together with one CoA-Ac-MDEL ligand (with the peptide part M1_p_D2_p_E3_p_L4_p_) for both protein chains. The root mean square deviation (rmsd) between the two protein molecules is 0.2 Å (for 190 Cα), indicating a very low level of flexibility.

**Table 1:** Data collection and refinement statistics (molecular replacement).

	*Ct*Naa20/CoA-Ac-MDEL
Data collection	
Space group	P 1 2_1_ 1
Cell dimensions	
* a, b, c* (Å)	46.2, 114.5, 47.4
* α, β, γ* (°)	90, 90.2, 90
Resolution (Å)	47.42–1.57 (1.60–1.57)^a^
R_merge_	0.087 (1.306)
I/σ(I)	8.6 (1.4)
Completeness (%)	98.7 (98.4)
Redundancy	6.6 (6.9)
Refinement	
Resolution (Å)	47.42–1.57 (1.60–1.57)
No. reflections	65018 (6699)
R_work_/R_free_ (%)	17.1/20.6
No. atoms	
Protein	3196
Ligand	102
Water	577
B-factors (Å^2^)	
Protein	29.1
Ligands	28.0
Water	40.6
R.m.s deviations	
Bond lengths (Å)	0.011
Bond angles (°)	1.035

^a^Values in parenthesis are for the highest-resolution shell.

The structure was determined from one crystal.

The *Ct*Naa20 structure consists of 4 α-helices and 8 β-strands and the bisubstrate is bound in the known V-shaped binding groove typical for NATs^30,33,41,42^. The β8-strand is a short additional strand compared to other NATs and shows that *Ct*Naa20 C-terminal residues fold back along the β6 strand (Fig. 4a). This interaction stabilizes the enzyme compared to a C-terminally truncated version *Ct*Naa20_1-166_ (Supplementary Fig. 4f).

Comparison of our structure with the *Ca*Naa20 part of *Ca*NatB shows that both catalytic subunits superimpose very well with a rmsd of 1.0 Å (for 158 Cα)^30^, although one is in complex with its auxiliary subunit and the other not (Fig. 4b). The nicely resolved *Ct*Naa20 C-terminal region superimposes well with the only partially built *Ca*Naa20 C-terminal region (Fig. 4b). The comparison shows that the Naa20 substrate peptide binding site does not undergo major conformational changes upon complex formation, and explains the ability of *Ct*Naa20 to bind and acetylate MDEL without *Ct*Naa25. Nevertheless, some loop regions differ. For example, the β3-β4 loop is longer in *Ct*Naa20 and does not superimpose well with the *Ca*Naa20 β3-β4 loop. The *Ct*Naa20 β6-β7 loop folds over the substrate peptide and contacts the α1-α2 loop, while the *Ca*Naa20 β6-β7 loop turns away from the peptide (Fig. 4a, b). This results in a narrower peptide binding site in *Ct*Naa20 compared to *Ca*Naa20. Taken together, we obtained a high-resolution crystal structure of *Ct*Naa20 alone, which superimposes well with *Ca*Naa20 as part of the *Ca*NatB complex, but also shows minor differences.

### *Ct*Naa20 structure explains the substrate specificity

The *Ct*Naa20 structure demonstrates how the catalytic subunit binds a substrate peptide in the absence of *Ct*Naa25. The loops α1-α2 and β6-β7 and the elongated β3-β4 loop confine the size of the substrate entry site. Loops α1-α2 and β6-β7 fold over the peptide, whereas loop α3-α4 is positioned underneath the peptide, but does not contact it directly (Fig. 4a). There are specific interactions between the ligand and *Ct*Naa20, which were also observed in the *Ca*NatB structure^30^. The acetyl bridge of the bisubstrate is coordinated by the amide backbone of V84. The amide nitrogen of M1_p_, the target of acetylation, is binding to the carbonyl oxygen of F118 (Fig. 4c and Supplementary Fig. 5a). The sidechain of M1_p_ is situated in a pocket built by Y27 and A83 with a contribution of T24 and E25 sidechains (Fig. 4c and Supplementary Fig. 5a). Noteworthy, this pocket is less hydrophobic compared to methionine pockets of other NATs, like Naa50 or Naa60, which also act on the initiator methionine^33,41,45^. The M1_p_ backbone carbonyl is bound by the hydroxyl group of Y145 and the D2_p_ amide is coordinated by the T82 carbonyl group (Supplementary Fig. 5a). The specificity for acidic residues in peptide position two is caused by the H80 and T28 sidechains, which both form hydrogen bonds to the D2_p_ sidechain (Fig. 4c). The D2_p_ backbone carbonyl binds to the Y27 sidechain and the amide of E3_p_ hydrogen bonds to Y144 sidechain (Supplementary Fig. 5a). The E3_p_ peptide sidechain hydrogen bonds to the G146 backbone (Supplementary Fig. 5a), but no sidechain specific protein-ligand interactions are found for E3_p_ and L4_p_, highlighting that the substrate specificity is mainly determined by the first two positions. Noteworthy, a well-ordered water can be found in the active site, which may be involved in catalysis. This water is coordinated by the backbone of F118 and I81, the M1_p_ amide and the D2_p_ sidechain, (Supplementary Fig. 5b).

Besides, the electrostatic surface potential of *Ct*Naa20 reveals a positive area at the conserved AcCoA binding site, but no further exposed positive patches (Supplementary Fig. 5c), corroborating the lack of *Ct*Naa20 binding to RNAs (Fig. 1e and Supplementary Fig. 2). In summary, the structural data support the observation that *Ct*Naa20 acetylates canonical NatB substrates in vitro and show that H80 is important for specific peptide binding.

### Specific residues are crucial for *Ct*NatB activity

**Fig. 5:**
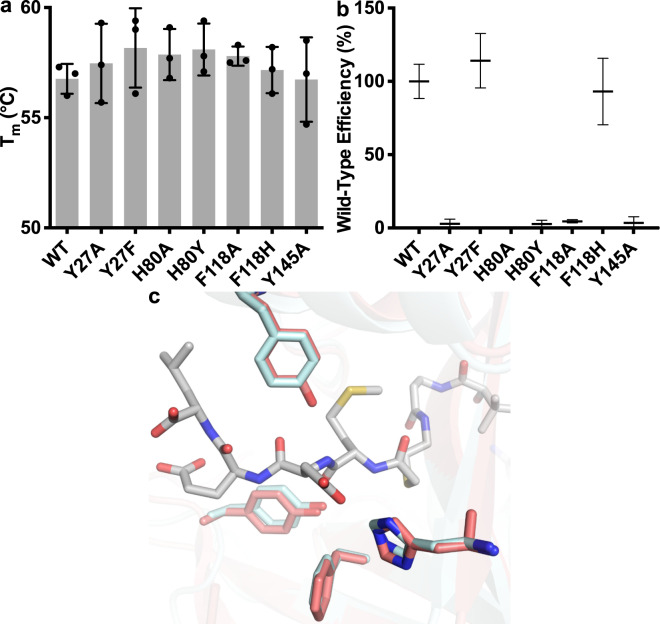
Characterization of *Ct*NatB mutants. **a** The stability of different NatB mutants (mutations in the catalytic subunit *Ct*Naa20_1-166_) was assessed by nanoDSF in triplicates and error bars represent the standard deviations. **b** The wild-type efficiencies of different point mutants were calculated from k_cat_ and K_m_ values, determined in individuals experiments in triplicates. The data represent the mean values with standard deviations of these experiments, normalized to the wild-type value and considering the propagation of uncertainty. **c** Superimposition of mutated residues with the corresponding *Ca*Naa20 residues. *Ct*Naa20 is shown as transparent cartoon with catalytically important residues shown as red sticks. The corresponding *Ca*Naa20 residues (pdb: 5k18^30^) are shown as cyan sticks and CoA-Ac-MDEL is shown as gray sticks.

To further investigate the enzymatic mechanism of *Ct*NatB, we mutated residues in *Ct*Naa20 which are suggested to be important for acetylation efficiency^30^. Single mutations in the *Ct*Naa20 substrate-binding pocket and active site (Y27A, H80A, H80Y, F118A, and Y145A) do not affect protein stability (Fig. 5a), but impair *Ct*NatB acetylation efficiency (Fig. 5b). When *Ct*Naa20 Y27 was replaced by F, which is the corresponding residue in *Ca*Naa20 (Supplementary Fig. 1b), or F118 replaced by H, the corresponding residue in Naa10, Naa50 and Naa60 (Supplementary Fig. 6a), the catalytic efficiency of the resulting *Ct*NatB complex does not change (Fig. 5b). Noteworthy, the H80Y and F118H *Ct*NatB mutants and the corresponding double mutant, which were created to mimic the Naa10, Naa50 or Naa60 sequences at these positions (Supplementary Fig. 6a), are not sufficient to alter the substrate specificity of NatB to accept SESS, EEEI or MVNAL peptides (Supplementary Fig. 6b). To test whether *Ct*Naa20 H80 is the major determinant for Naa20 and NatB substrate specificity (Fig. 4c), we created the *Arabidopsis thaliana* Naa60 Y115H mutant, to mimic the Naa20 sequence at the corresponding position (Supplementary Figs. 1b and 6a). This Naa60 mutant acetylated the NatB substrate MDEL in addition to its canonical substrates (Supplementary Fig. 6c), while the *At*Naa60 wild-type is not active towards MDEL^41^. This confirms that H80 is indeed a key residue for Naa20 substrate specificity.

The inactive *Ct*Naa20 mutants (Y27A, H80A, H80Y, F118A, and Y145A) were also tested for CoA-Ac-MDEL binding using nanoDSF (Supplementary Fig. 7). The stabilizing effect of CoA-Ac-MDEL is higher than that of AcCoA for the Y27A and F118H mutant, indicating that they still bind MDEL (Supplementary Fig. S7a, e). In contrast, the H80A, H80Y, F118A, and Y145A mutants are not able to bind MDEL (Supplementary Fig. 7b–f) explaining their acetylation deficiency. Comparison with *Ca*Naa20 shows that the corresponding residues superimpose well (Fig. 5c), which implies that their role in peptide binding and substrate acetylation is conserved between Naa20 proteins. Taken together, our data show that *Ct*Naa20 alone is active in substrate acetylation and highlight the importance of specific residues for peptide binding and acetylation activity, and suggest that Naa25 binding does not induce conformational changes in Naa20.

## Discussion

The majority of the proteome is N-terminally acetylated with around 20% being acetylated by NatB in yeast, plants, and human^46^. However, compared to the major NatA/NatE complexes, NatB has been less studied. For NatA and NatE, structures of the complexes^32,42,47^ and of the individual catalytic subunits Naa10 and Naa50^32,33^ were reported and analyzed in depth. Recently, the *Ca*NatB crystal structure and the *Hs*NatB cryo-EM structure were determined^30,31^. This showed how Naa25 binds to Naa20, and provided the first molecular basis of NatB substrate specificity. However, the Naa20 subunit could not be purified alone in previous *Ca*NatB and *Arabidopsis thaliana* (*At*) NatB studies^23,30^. Therefore, to characterize NatB in more detail, we aimed to investigate *Ct*Naa20 structure and function in the absence of *Ct*Naa25.

Both *Ct*NatB subunits were expressed and purified independently to homogeneity, and were shown to be monomers in solution. A stable *Ct*NatB complex was formed with a 1:1 stoichiometry and a K_d_ of 17.8 ± 11.9 nM. Comparison with NatA binding to Naa50 (K_d_ = 46 ± 8.8 nM)^48^ shows that the binding affinities are in the same range. *Ct*NatB acetylates specifically the MDEL peptide with enzymatic parameters similar to the ones described for other organisms. This emphasizes a high degree of conservation of NatB substrate specificity and mode of action^3,23,30,49^. Surprisingly, we also observed that *Ct*Naa20 alone specifically acetylates the MDEL peptide in vitro, but with a lower efficiency than *Ct*NatB. So far, Naa20 was considered to be inactive without Naa25 in vivo^15,16,24^ and the in vitro activity was not tested due to the lack of stable Naa20. In order to understand the seeming discrepancy between in vivo inactivity and the in vitro activity described in this study, we determined the *Ct*Naa20 RNA binding capability and analyzed the *Ct*Naa20 electrostatic surface potential. *Ct*Naa20 does not bind RNA and does not present exposed positive patches that would allow for a direct ribosome interaction (Fig. 1e and Supplementary Fig. 5c). Therefore, Naa20 alone most likely does not bind to the ribosome and might have limited access to its substrates, which could explain the in vivo inactivity of Naa20. For comparison, Naa10 does not expose positive patches and is not involved in NatE-ribosome interactions^36^. Noteworthy, Naa20 is also found in the nucleus as a single subunit^13^ and thereby might acetylate a subset of presumed NatB substrates post-translationally. Interestingly, NatB-type substrates are found overrepresented in the nucleus compared to the whole worm lysate in *C. elegans* and a higher acetylation rate is observed in the nuclear fraction^12^.

The observed differences in *Ct*NatB and *Ct*Naa20 acetylation efficiency is reminiscent of differences in the activity towards the MLGP peptide between human Naa50 alone and as part of the human NatE complex^48^. In this case, *Hs*Naa50 in complex with *Hs*NatA (forming *Hs*NatE) increased its affinity for the peptide, which lead to a more efficient acetylation. Similarly, we showed by enzymatic measurements that one function of Naa25 is to increase the affinity of Naa20 for NatB substrates. Probably, this is due to a stabilizing effect of *Ct*Naa25 on *Ct*Naa20, which leads to a more efficient acetylation.

Based on our results, showing that *Ct*Naa20 specifically acetylates the MDEL peptide, we designed and synthesized the CoA-Ac-MDEL bisubstrate analog. This ligand was used for co-crystallization with *Ct*Naa20 and structure determination at 1.57 Å resolution. The structure with the CoA-Ac-MDEL ligand allowed understanding the observed substrate specificity for the MDEL peptide. The M1_p_ sidechain of the ligand is located in a wide pocket and importantly the D2_p_ sidechain is coordinated by the H80 sidechain. Compared to other catalytic subunits acting on the initiator methionine, like Naa50 or Naa60, this specific sidechain interaction with the substrate residue in position two is a unique feature of Naa20 and of NatB^30^. The responsible histidine sidechain is conserved in all Naa20 subunits (Supplementary Fig. 1b) and explains the specificity for acidic residues at substrate position two. Compared to other NATs, Naa10 also has a conserved corresponding histidine, which is however not involved in substrate binding^42^. Interestingly, an *At*Naa60 Y115H mutant was sufficient to broaden Naa60 substrate specificity towards NatB substrates, highlighting that this histidine plays a central role in recognition of an acidic residue in substrate position two. Naa30, Naa50, or Naa60 have a tyrosine at this position (Supplementary Fig. 6a). Nevertheless, a single H80Y mutation in *Ct*Naa20 was not sufficient to change the substrate specificity to NatC/E/F like substrates. The *Ct*Naa20 methionine binding pocket is more hydrophilic compared to Naa50 and Naa60^33,41^, consistent with Naa20 specificity for polar residues in position two. The substrate peptide residues E3_p_ and L4_p_ are not involved in specific protein-ligand interactions and may play only a minor role in substrate recognition. Accordingly, the third and fourth position of NatB substrates was found to be highly variable^3,23,49^.

When compared with the *Ca*Naa20 subunit in complex with *Ca*Naa25, *Ct*Naa20 alone superimposes very well and only differs in several loop regions. The *Ct*Naa20 β3-β4 loop is longer compared to *Ca*, and the β6-β7 loop folds over the MDEL peptide and is in closer contact to loop α1-α2 than in *Ca*. The high similarity of both structures suggests that Naa25 does not induce major rearrangements in Naa20 and that the mechanism of substrate binding and acetylation is very similar between NatB and the isolated Naa20 subunit. Similarly, binding of *Ca*Naa20 seems not to induce conformational changes in the *Ca*Naa25 subunit^30^. Here, NatB differs distinctly from NatA, as the peptide-binding pocket of Naa20 remains unchanged upon NatB complex formation and Naa20 is catalytically active on its own. Formation of NatA induces rearrangements of catalytically important residues in Naa10 and alters its substrate specificity as Naa10 alone is not active towards NatA substrates^32^. The *Ct*Naa20/CoA-Ac-MDEL structure supports the observation that *Ct*Naa20 is active towards canonical NatB substrates in vitro. Nevertheless, we cannot exclude that *Ct*Naa20 is also active towards other substrates in the absence of *Ct*Naa25.

Our structure unravels the Naa20 peptide binding mode, but does not allow to deduce an exact catalytic mechanism. In general, NATs were shown to use a base-mediated mechanism. Naa10 uses a distinct glutamate as general base and the corresponding E25 of *Ct*Naa20 is conserved in Naa20 proteins (Supplementary Figs. 1b and 6a)^32^. However, in the *Ct*Naa20 structure, E25 is not positioned in a way to serve as a base and the corresponding E25A mutation in the *Ca*NatB complex even increased the catalytic efficiency^30^. Naa50 and Naa60 were shown to use a dual-base mechanism with conserved tyrosine and histidine residues and a well-ordered water molecule^33,41^. A well-ordered water was also found in the active site of the *Ct*Naa20 structure. This water is likely to be involved in the catalysis, however, a basic residue is not involved in the coordination of the water and therefore a definite catalytic mechanism cannot be deduced. In *Ct*Naa20, H80 corresponds to the catalytically important tyrosine of Naa50/60 and is important for binding D2_p_, but H80 cannot additionally hydrogen bond to the active site water or the M1_p_ amide. The *Ct*Naa20 residue corresponding to the catalytically important histidine of Naa50/60 is F118, which again is crucial for peptide binding and involved in coordinating the active site water, but is not a basic residue and therefore cannot be involved in proton transfer. Additionally, the F118H mutant showed no change in acetylation efficiency, whereas the corresponding H154F mutation in Naa60 impaired activity^41^. However, one has to consider that the bisubstrate analogs used here and in previous structures do not reflect the accurate transition state geometry of N-terminal acetylation, which proceeds via a tetrahedral conformation of the transferred acetyl-group carbon. This different geometry may hinder the identification of the *Ct*Naa20 catalytic base, or the exact role of the active site water. Nevertheless, the CoA-Ac-MDEL bisubstrate analog is a potent competitive NatB inhibitor and a useful tool to understand the binding mechanism, structural features, and function of NATs. As NAT dysregulation is linked to multiple human diseases^9^, it is momentous to design NAT inhibitors for therapeutic purposes, based on substrate specificities and available structures, and to design ways to synthesize bio-available inhibitors in the future. NatB is an interesting therapeutic target because its depletion leads to the most severe phenotypes among all NATs^50^.

Taken together, our data contribute to a detailed understanding of NAT structures and functions, by providing the first Naa20 crystal structure. We show how Naa20 binds and specifically acetylates its substrates, which indicates that Naa20 may have a function without Naa25. Therefore, our study provides the structural and mechanistic framework to fully integrate NatB into the landscape of N-terminal protein acetylation.

## Methods

### Cloning of NatB constructs

*CtNAA25* was amplified by PCR from cDNA and an internal NcoI-site was abolished by introducing a silent mutation in the forward primer. The resulting fragment was digested with the NcoI and BamHI enzymes and ligated into the pETNHis-vector (G. Stier, BZH) to obtain the pETNHis::*CtNAA25* construct with a TEV-site cleavable His_6_-tag. *CtNAA20* was amplified from cDNA by PCR and transferred into the TOPO-vector (Thermo Fischer). One internal NcoI-cutting site was removed by site-directed mutagenesis and a C-terminal His_6_-tag was introduced. The *CtNAA20* fragment was ligated into the pET21d vector, leading to the pET21d::*CtNAA20*-His construct. Finally, the C-terminally truncated construct pET21d::*CtNAA20*_1-166_-His was obtained by PCR using the full-length construct as template and cloning into the pET21d vector. Different *CtNAA20*_1-166_ point mutants were obtained by site-directed mutagenesis using the quikchange lightning kit according to the manufacturer protocol (Agilent). All used primers are listed in Supplementary Table 1.

### Expression and purification of native proteins

The pET21d::*CtNAA20*His construct was expressed in Rosetta II (*DE3*) *E. coli* strain (Novagen). Cells were grown at 18 °C in auto-induction-media, supplemented with chloramphenicol (34 μg ml^−1^) and ampicillin (50 μg ml^−1^). For purification, the cells were resuspended in lysate buffer (20 mM HEPES pH 7.5, 200 mM NaCl, 40 mM Imidazole), supplemented with a protease inhibitor mix, lysed using a microfluidizer (M-110L, Microfluidics) and the lysate was cleared by centrifugation (50000 × *g*, 25 min, 4 °C). For Ni-IMAC (immobilized metal affinity chromatography), the supernatants were loaded on a 1 ml HisTrap HP column (GE Healthcare). The tagged protein was eluted by adding 250 mM imidazole to the lysis buffer and further purified by size exclusion chromatography (SEC) using a S75 16/60 gel-filtration column (GE Healthcare) and buffer G (20 mM HEPES pH 7.5, 200 mM NaCl).

For the purification of the *Ct*Naa25 subunit, the *Ct*NatB complex or the complex with Naa20_1-166_ point mutants, a three-step purification was performed. For this, the pETNHis::*CtNAA25* construct, was transformed alone or cotransformed with the pET21d::*Ct*NAA20-His, or one of the *CtNAA20*_1-166_-His point mutants, with Rosetta II (*DE3*) *E. coli* cells (Novagen). Cells were grown at 18 °C for 18 h in auto-induction-media, supplemented with chloramphenicol (34 μg ml^−1^) and kanamycin (50 μg ml^−1^) for the *Ct*Naa2*5* expression and additionally with carbenicillin (50 µg ml^−1^) for the different *Ct*NatB complex species. For purification, the cells were resuspended in lysate buffer, supplemented with a protease inhibitor mix, lysed using a microfluidizer (M-110L, Microfluidics) and the lysate was cleared by centrifugation (50000 g, 25 min, 4 °C). The proteins were purified with Ni-IMAC by loading the supernatant on two 1 ml HisTrap HP column (GE Healthcare). Elution was performed with 250 mM imidazole. Afterward, the samples were dialyzed against IEX buffer A (100 mM NaCl and 50 mM sodium citrate pH 5.5) and loaded on a 5 ml HiTrap SP column for cation-exchange chromatography. The proteins were eluted using IEX buffer B (50 mM sodium citrate pH 5.5 and 1100 mM NaCl) by applying a step gradient of 18% buffer B. Afterwards the buffer was exchanged to buffer G via dialysis and the sample was loaded on a Superdex 200 26/60 gel-filtration column (GE Healthcare) for SEC. The *At*Naa60_Y115H mutant was expressed and purified as described recently^41^.

### Crystallization of *Ct*Naa20

Crystallization was performed at 18 °C using the sitting drop vapor diffusion method. *Ct*Naa20 was concentrated after gel-filtration to 20 mg/ml and mixed in a 1:3 molar ratio with CoA-Ac-MDEL and incubated on ice for 18 h. The crystallization drops contained 200 nl protein solution and 200 nl precipitant solution (15% (v/v) propanol, 0.2 M ammonium acetate, and 0.1 M TRIS pH 8.5). Crystals appeared after 3 days and were cryo-protected with 20% glycerol and flash-frozen in liquid nitrogen.

### Data collection and structure determination

Data sets for the *Ct*Naa20 crystals were collected at beamline P14 (DESY) at cryogenic temperature. The images were integrated with XDS^51^. Afterwards the images were scaled using AIMLESS^52^. Phases were obtained by molecular replacement with PHASER-MR^53^ implemented in the PHENIX package^54^. The *Ca*Naa20 part of the *Ca*NatB complex (^30^ pdb:5k18) was used as an initial search model. Finally, iterative model building and refinement were performed with Coot^55^ and Phenix.refine^56^. The CoA-Ac-MDEL ligand was parametrized with the PHENIX eLBOW module in AM1 QM mode^57^. Model quality was analyzed with MolProbity^58^. Interfaces and crystal packing were analyzed with PISA^59^. Structure figures were prepared with PyMOL^60^. Crystallographic data are summarized in Table 1. Coordinates and structure factors are deposited at the Protein Data Bank PDB with accession code 6ZMP.

### SEC-MALS analyses

The *Ct*NatB complex (0.12 mg) and its subunits *Ct*Naa25 (0.13 mg) and *Ct*Naa20 (0.1 mg) were successively injected onto a Superdex 200 10/300 gel-filtration column (GE Healthcare) in buffer G. The column was connected to a MALS system (Dawn Heleos II 8+ and Optilab T-rEX, Wyatt Technology). Data were analyzed using the Astra 6 software (Wyatt Technology).

### RNA preparation and electrophoretic mobility shift assay

The 32 nucleotides long (5′-GGGCCTCTAGCCGGGCAACCGGCCGGCGGCTC-3′) *Ct*ES27 fragment of the 25 S rRNA fused to the 3′-hammerhead ribozyme was amplified by primer extension PCR and cloned under the control of the T7 RNA polymerase promotor into the pUC18 vector (Promega) digested EcoRI/ Hind III. In vitro transcription of the *Ct*ES27 fragment and hammerhead ribozyme RNA construct was performed as described previously^61^. In brief, the *Ct*ES27 fragment RNA and hammerhead ribozyme RNA were transcribed in vitro and purified by urea-polyacrylamide gel electrophoresis, extracted by crush-and-soak followed by isopropanol precipitation and desalting.

For the EMSA, *Ct*Naa20 and *Ct*NatB (5–20 µM) were incubated with 5 µM *Ct*ES27 or hammerhead ribozyme RNA in EMSA buffer (20 mM Tris pH 8, 200 mM NaCl, 10 mM MgCl_2_, 10 mM KCl and 20% v/v glycerol) at 20 °C for 30 min. The samples were separated electrophoretically with 0.8% (w/v) agarose gels (supplemented with ethidium bromide) in 0.5x TB buffer (45 mM boric acid and 45 mM Tris pH 8). RNA-free and protein-free samples were used as controls. The RNA migration was visualized under UV light.

### Synthesis of CoA-Ac-MDEL inhibitor

For the synthesis of the bisubstrate analog CoA-Ac-MDEL, the Foyn et al. protocol was modified^43^. The MDEL peptide was synthesized with a MultiPep RSi peptide synthesizer (Intavis) on solid support. A leucine preloaded 2-chlorotrityl resin (50 µmol, 1 equiv.) was used with 9-Fluorenylmethoxycarbonyl (Fmoc)-amino acids (250 µmol, 5 equiv), HBTU (2-(1H-benzotriazol-1-yl)-1,1,3,3-tetramethyluroniumhexafluoro-phosphate; 250 µmol, 5 equiv), HOBt (1-Hydroxybenzotriazol; 0.2 mol/l) and DIPEA (Diisopropylethylamine; 500 µmol, 10 equiv) in DMF (*N,N*-Dimethylformamide) doing double couplings for 40 min. Fmoc deprotection was carried out applying 40% piperidine in DMF for 3 min and then 20% piperidine in DMF for 14 min. 70 mg Bromoacetic acid (500 µmol, 10 equiv), dissolved in DMF and mixed with 155 µl DIC (*N*,*N*’-diisopropylcarbodiimide; 126 mg, 1 mmol, 20 equiv) was added to the peptide-linked resin. The suspension was shaken for 24 h at room temperature and then the resin was washed three times with DMF and DCM (Dichloromethane), respectively. The bromo-acetylated peptide was cleaved from the resin by gently shaking in a cleavage cocktail (Trifluoroacetic acid:Triisopropylsilane:water 95:2.5:2.5) for 3 h and was precipitated in 40 ml cold diethylether and dried under vacuum. The precipitate was dissolved in 2 ml water/acetonitrile mixture (80:20) and purified by reverse phase HPLC. The solvent was removed using a rotary evaporator revealing a white solid (3.5 mg, 5.6 µmol, 11.2% yield). Subsequently, the intermediate was dissolved in 200 µl of 1 M triethylammonium bicarbonate buffer at pH 8.5 and a 200 µl solution of 6.4 mg Coenzyme A trilithium salt (8.49 µmol, 1.5 eq.), in the same buffer, was added. The mixture was stirred at room temperature for 24 h protected from light. The solvent was removed under vacuum and the crude product was dissolved in water and purified by reverse phase HPLC (water/acetonitrile 10–30%) to a purity of >95%. The solvent was removed by lyophilization to give 0.9 mg of CoA-Ac-MDEL (0.7 µmol, 12.5% yield for CoA coupling; MALDI/TOF (pos): m/z calc. for C_43_H_71_N_11_O_26_P_3_S_2_^+^: 1314.32 [M + H]^+^; found: 1314.2).

### *Ct*NatB activity and CoA-Ac-MDEL inhibition assays

All enzymatic assays (the substrate specificity tests of *Ct*NatB and *Ct*Naa20, the Michaelis–Menten analysis of the *Ct*NatB complex and the complex with the mutated catalytic subunits, the inhibitor assays to determine the IC_50_ value of CoA-Ac-MDEL and the mode of inhibition) were performed using microplate assays described earlier^62^ and used as modified recently^41^. For all assays a protein concentration of 500 nM was used. For all assays, either a constant peptide concentration of 1.5 mM or a constant AcCoA concentration of 370 µM with varying concentrations of 6–500 µM AcCoA or 39–2500 µM MDEL were used. Background control reactions were performed in the absence of the enzyme, or of the peptides and all reactions were performed in triplicates. Data were evaluated using the GraphPad Prism software.

### ITC Measurements

ITC binding measurements between *Ct*Naa25 and *Ct*Naa20_1-166_ were performed using a PEAQ-ITC microcalorimeter (Malvern Instrument GmbH). Prior to the measurements, the protein samples were dialyzed against buffer G overnight.

*Ct*Naa20_1-166_ concentrations of 25–35 µM in the cell were titrated with *Ct*Naa25 concentrations of 250–400 µM in the syringe at 20 °C. The data were fitted and analyzed using a single-site binding model in the MicroCal PEAQ-ITC analysis software. Measurements were performed in triplicates. In addition, buffer to buffer, buffer to *Ct*Naa20_1-166_ and *Ct*Naa25 to buffer ITC runs were performed as control reactions.

### NanoDSF Measurements

To determine melting temperatures T_m_ of different protein samples, nano differential scanning fluorimetry (nanoDSF) was used. Intrinsic tyrosine and tryptophan fluorescence at emission wavelengths of 330 nm and 350 nm were measured continuously applying a temperature gradient of 20–90 °C in the Prometheus NT.48 nanoDSF system. The T_m_ was calculated by the supplied software (NanoTemper Technologies GmbH). To assess the stability of the different *Ct*NatB mutants, 1 mg/ml samples were measured in buffer G. The stability changes of *Ct*NatB, *Ct*Naa20, and its mutants upon addition of different ligands, were measured using 15–30 µM protein in buffer G, after incubation with a varying excess of AcCoA, CoA-Ac-MDEL, CoA-Ac-SESS, or CoA-Ac-MVNAL for 10 min on ice.

### Statistics and Reproducibility

All kinetic experiments were performed in triplicates. Error bars in figures represent the standard deviations. All nanoDSF assays were performed in triplicates or quadruplicates and error bars represent the standard deviations. Individual data points are depicted in all figures, apart for Fig. 5b. For Fig. 5b, k_cat_ and K_m_ values of each mutant were determined in individuals Michaelis-Menten experiments in triplicates, which were further used to calculate the enzymatic efficiency, normalized to the wild-type efficiency. The data represent the mean values with standard deviations considering the propagation of uncertainty. The ITC measurements were performed in triplicates and K_d_ and ΔH represent the mean values with corresponding standard deviations.

## Supplementary information

Peer Review File

Supplementary Information

Description of Additional Supplementary Files

Supplementary Data 1

## Data Availability

Coordinates and structure factors have been deposited at the Protein Data Bank under the accession code 6ZMP. All source data underlying graphs and charts are presented in Supplementary Data 1. Further data supporting the findings of this study are available from the corresponding author upon reasonable request.
