# Supplementary material for: Structural basis of Naa20 activity towards a canonical NatB substrate
Source: Commun Biol. 2021 Jan 4;4:2. doi: 10.1038/s42003-020-01546-4 (PMC7782713; doi:10.1038/s42003-020-01546-4)
Supplement: Supplementary file 2 — Supplementary Information [file 42003_2020_1546_MOESM2_ESM.pdf]

Supplementary Information

Structural basis of Naa20 activity towards a canonical NatB substrate

Dominik Layer et al.

a

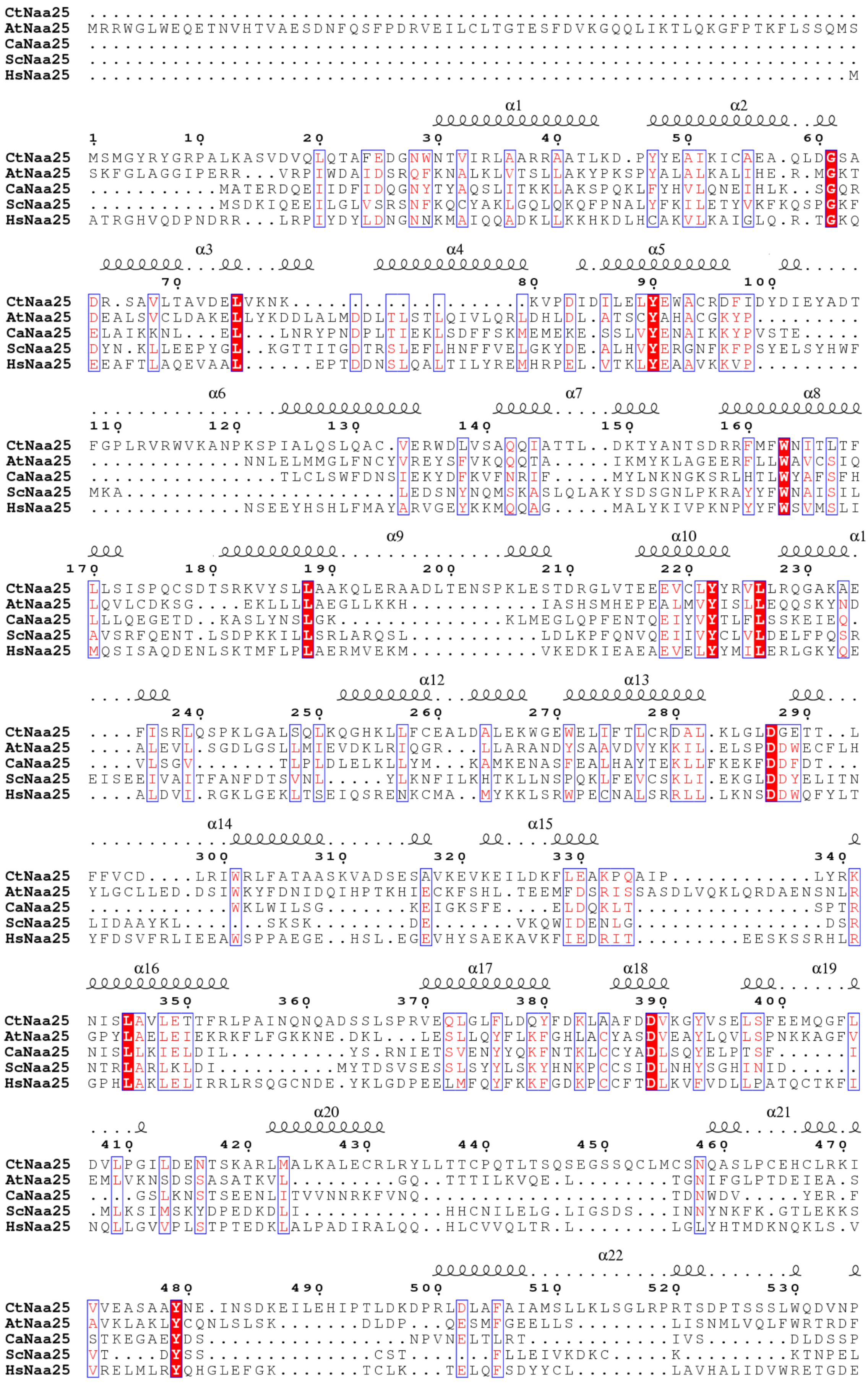
CtNaa25
AtNaa25
CaNaa25
ScNaa25
HsNaa25
α1
α2
α3
α4
α5
α6
α7
α8
α9
α10
α11
α12
α13
α14
α15
α16
α17
α18
α19
α20
α21
α22

```
        α23                     α24                 α25           α26
        540         550         560         570         580         590         600
CtNaa25 GLFLQAVLLLDSQLKFTPNNVELRLLLVQLYLLLGCASYAYQLWNPLGVKRTIQDALSPLFFDRI
AtNaa25 GYLAEAIMVLELGLTIRGHVWQYKILLLHIYSYVGALPLAFERYKALDVKNILTETVSHHILRQM
CaNaa25 QNTIKNIVLLKHLLEQDKYNYKLKLWLMKLYSQLNTNDLIFPIYNGLKIRMTQHETLNYYLTTTN
ScNaa25 KDVLLCITILENYQAKDPHNFDTMCWLIVLYMYLGLVPDAYFHFINLKIKNVQTDSLDYMIFSRF
HsNaa25 TTVWQALTLLEEGLTHSPSNAQFKLLLVRIYCMLGAFEPVVDLYSSLDAKHIQHDTIGYLLTRYA

                      α27                          α28
        610         620         630         640         650         660
CtNaa25 SS.LSPGLFQGSVPPT.EKLRSFYNQSLRDNSPVRIWDAFLAGSYTSILDMSEYDSRLRRSCTLM
AtNaa25 LE...SPMWVDLSNLL.KDYLKFMDDHLRESA.DLTFLAYRHRNYSKVIEFVLFKQRLQHSNQYQ
CaNaa25 PSKINLDAWV........DIYRFYLTSKQ.EIKESIIQGFDNGVFNKLEGFINFSKRMQNSISLN
ScNaa25 ST.LFPNKQSDFYSKTFHEHNNLYDTS.LANLPRYIQVAFERNSYSKILGMLEMRDKLMKSYTRW
HsNaa25 ES...LGQYAAASQSC.NFALRFFHSNQKDTS.EYIIQAYKYGAFEKIPEFIAFRNRLNNSLHFA

          α29                      α30           α31
          670         680         690         700         710
CtNaa25 MTVVEERRARRCYGARQ...........ECEIEAHPLLDKITHDTVLVDKTD.......YGPFPN
AtNaa25 AARVEASVLQLKQNADSFEEEERILENLKSGVQLVELSNEIGSRTL.KFNEDMQTRP.WWTPCPE
CaNaa25 FTVAKILQISTILGTDGYLNYF..IHYLK.......T.NEALIVSDYTDNRDFKS...EWNGLEK
ScNaa25 TKTLENLQFSRLCNDKRGHLLQKLHEDWR....SL....EMTQSVSFSDNRDFSILDENFAQFLN
HsNaa25 QVRTERMLLDLLLEANISTSLA...ESIK.SMNLRPEEDDIPWEDL.RDNRDLNVFF.SWDPK..

                  α32                   α33                α34
          720         730         740
CtNaa25 LESPHGPPIH........EYLRLGPGLSNER........SH...LAF......LAEQYMDL....
AtNaa25 KNYLLGPFEE.......ISYCPPKENVKEER........EENMKRAIQRKSLLPRMIYLSI..Q.
CaNaa25 IDCIDVPVNDVATKLKLLVYSIVFEDQDASRLLKVFNKITSNAKFSVFDN..LYKLYFNLLKI.
ScNaa25 R....GK..I.......LEYA.........................NLNEESIFLTL.......IR
HsNaa25 ..................DRDVSEEH........K...KLSLEEETLWLRIRSLTLRLIS

                   α35          α36                      α37
          750         760         770         780         790
CtNaa25 ..LSYKPPKDFKANR..PNEVAVRDREYTLDTLDD.......LSA......SLTDFINAPNTYS
AtNaa25 .....CTPTALK..E....SVETNGSGGDIDVCEELKCLLEDYTKMLGCSLSDAVEMITEIS...
CaNaa25 ....TKTKLNPQETQSLYNYLQKNLKTDKLKILIPEN....LLSGELTQNLTNLVEFIKIVK...
ScNaa25 ELIIEALPNGEKTEQ.................ISALLKKLP..................
HsNaa25 GLPSLNHPVEPKNSE....KTAENGVSSRIDILRL...LLQQLEATL....ETGKRFIEKDIQYP

                         α38
          800         810         820         830
CtNaa25 FLTPAERT.YYT........IISLLSSALFTALSSPRSEPVSKSLSSTT.................
AtNaa25 .QGARTSE.SLG......SNLVDWLNFAVFWNAWSLSSQE...HW.....................
CaNaa25 LLAKRHPSSYMNQLV...............NL..................................
ScNaa25 ......SI.NLEELLNNNLTEVESASFLIFFEIYENNGKNLHDLISRLMKVPINAKQNWMVSHTY
HsNaa25 FLGPV.PT.RMGGFFNSGCSQCQISSFYLVNDIYELDTSGLEDTM.EIQ................

                     α39                                        α40
                    840         850         860
CtNaa25 ..............ASIKKAFNSL.....R...........TTLSPTPNSPASLPALTPLTNLHT
AtNaa25 ..............HVLNSLFERLILDRVRSMGSSDMSSCYSDVQ....................
CaNaa25 ...........VKPFGKEFKNLKLVQRQ......................................
ScNaa25 LTKMATLKTLDSLKRIKDKEIQKLIKNSLK....ELRSCCDDVFKGYSK................
HsNaa25 ..............ERIENSFKS.LLDQLK...DVFSKCKGDLLEVKDG..NLK..THPTLLEN

        870         880         890         900         910         920         930
CtNaa25 LAFLRESALAIKHTSLFATSFHDKEISRDRTGRSGLHRDALSEMRVLGETATKALVELKGHVQKL
AtNaa25 .VLVQIITEPLAWHSLIIQACTRSSLPSGKKKKKNQHSDQLSSSPIS........QAIKDSIQLL
CaNaa25 .................HEII...............DSMDFEPPIS........VDISQTKLEI
ScNaa25 .AL......VQAYEEL.................KKDECGNLLKELDVKAE....NVKNIKNSLLGI
HsNaa25 LVFFVETISVILWVSSYCESVLRPYKLNLQKKKKKKETSIIMPPVF........TSFQDYVTGL

          α41
            940         950         960         970
CtNaa25 KEML.AEGGWLDRVLNL...........CFEKGG......DVEKAVEGVVGGREE..AEEWVGRV
AtNaa25 CSTIQDVSNWLLNQLNNPEDGQVEGFLTTLKRDGNAAGPGQILGVLESFIASSEESEVGNRIFQA
CaNaa25 KSSIEDCVVALLNSL...................................................
ScNaa25 QKSVRNL...........................................................
HsNaa25 QTLISNVV....DHIKGLET.....HLIALK..............LEELILEDTSLSPEERKFSK

        980         990
CtNaa25 VESWREGVKGWGMVRWE...........................
AtNaa25 LKSWNTADTARKTVMAQQRVLREFLQICESKRKLLETLKQQMSHV
CaNaa25 .............................................
ScNaa25 .............................................
HsNaa25 TVQ........G..KVQSSYLHSLLEMGELLKKRLETTKKLKI..
```

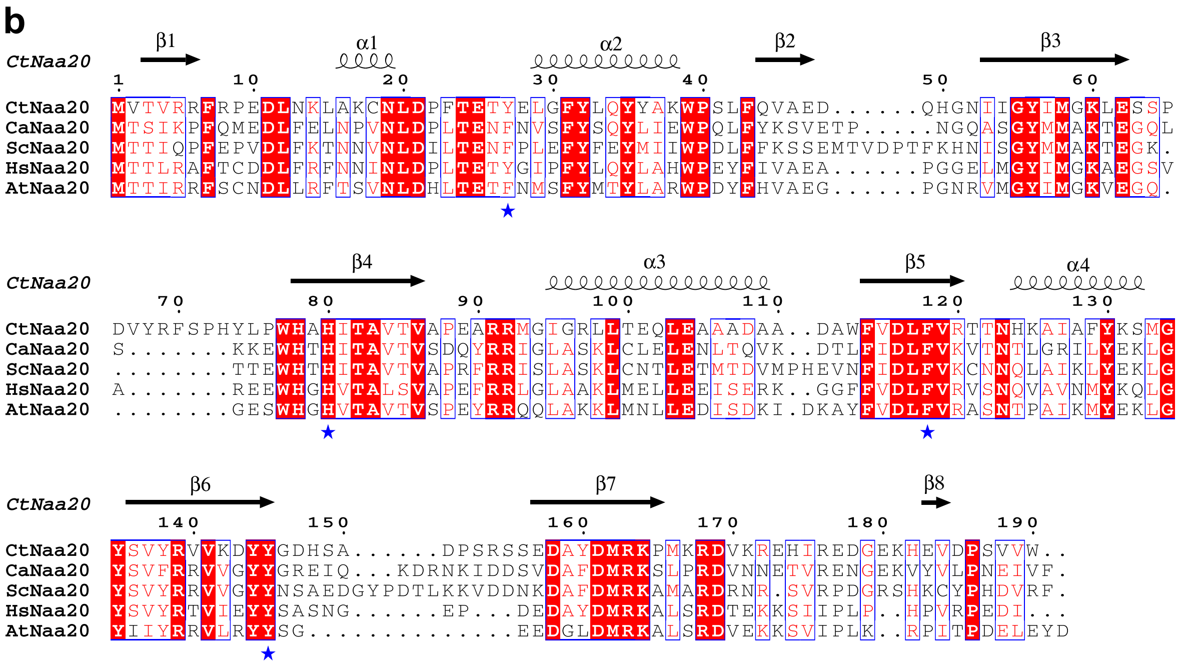

Supplementary Figure 1: Sequence alignment of Naa20 and Naa25 homologs. a: Sequence alignment of *Ct*Naa25, *At*Naa25, *Ca*Naa25, *Sc*Naa25 and *Hs*Naa25. α-helices of the *Ca*Naa25 structure are shown on top of the alignment. **b:** Sequence alignment of *Ct*Naa20, *Ca*Naa20, *Sc*Naa20, *Hs*Naa20 and *At*Naa20. The sequence alignments were performed using Clustal Omega and ESPript3[1,2]. Fully conserved residues are shown as white letters in red boxes and sequence similarities are shown with red letters in blue frames. α-helices and β-strands of the *Ct*Naa20 structure are shown on top of the alignment. Residues, which were mutated in this study are marked with a blue star. *Ct: Chaetomium thermophilum; Ca: Candida albicans; Sc: Saccharomyces cerevisiae; Hs: Homo sapiens; At: Arabidopsis thaliana.*

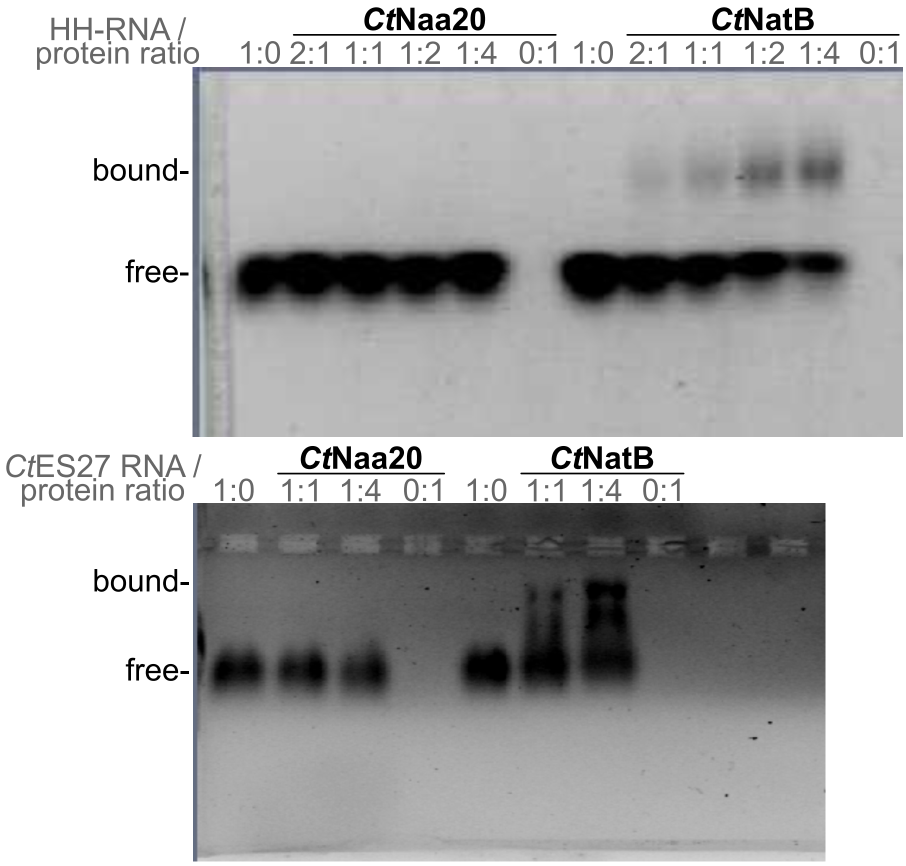

Supplementary Figure 2: Electrophoretic mobility shift assays. Electrophoretic mobility shift assays (EMSA) with hammerhead ribozyme or expansion segment 27 (ES27) RNA and *Ct*Naa20 or *Ct*NatB. The upper panel depicts the EMSA with hammerhead ribozyme RNA. *Ct*Naa20 is not binding, while *Ct*NatB is binding to the hammerhead RNA, indicating nonspecific RNA binding. The lower panel depicts the full gel of figure 1e. *Ct*Naa20 is not binding, while *Ct*NatB is binding to ES27 RNA.

a

	*Ct*NatB	*Ca*NatB[3]	*At*NatB[4]
K_m (μM)	45.6 ± 4.8	50.0 ± 8.0	38.4 ± 9.1
k_{cat} (min^{-1})	68.8 ± 2.0	63.6 ± 3.0	27.3 ± 1.6
k_{cat}/K_m (min^{-1} $μM^{-1}$)	1.50 ± 0.16	1.27 ± 0.21	0.71 ± 0.18

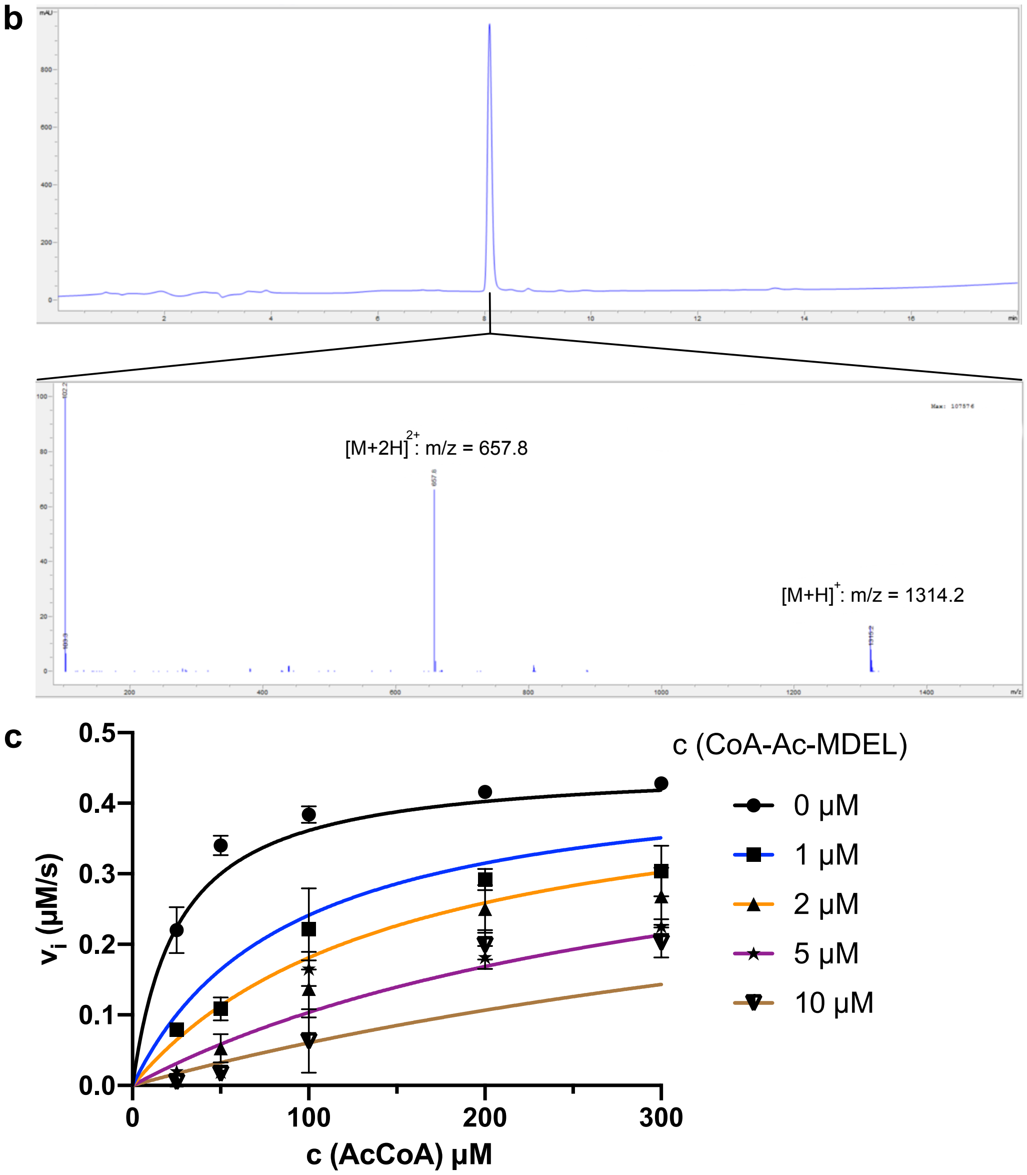

Supplementary Figure 3: Enzymatic parameters of *Ct*NatB and CoA-Ac-MDEL characterization. a: Kinetic constants of *Ct*NatB compared to the corresponding values of *Ca*NatB[3] and *At*NatB[4]. **b:** HPLC/MS of CoA-Ac-MDEL. The analytical HPLC profile of CoA-Ac-MDEL with one peak at a retention time of 8.1 min, indicating a high purity of the synthesized inhibitor is shown with the corresponding mass spectrum. The calculated m/z ratio for $[CoA\text{-}Ac\text{-}MDEL+H]^+$ is 1314.32 and the found m/z ratio is 1314.2. **c:** Inhibition assay to determine the mode of inhibition. Michaelis-Menten plots of the *Ct*NatB mediated acetylation of MDEL at different inhibitor concentrations (0-10 μM) were determined using various AcCoA concentrations (25-300 μM). The inhibitor constant K_i was calculated using GraphPad Prism (version 8.4.3) applying a competitive inhibition model. Measurements were performed in triplicates and error bars represent SD.

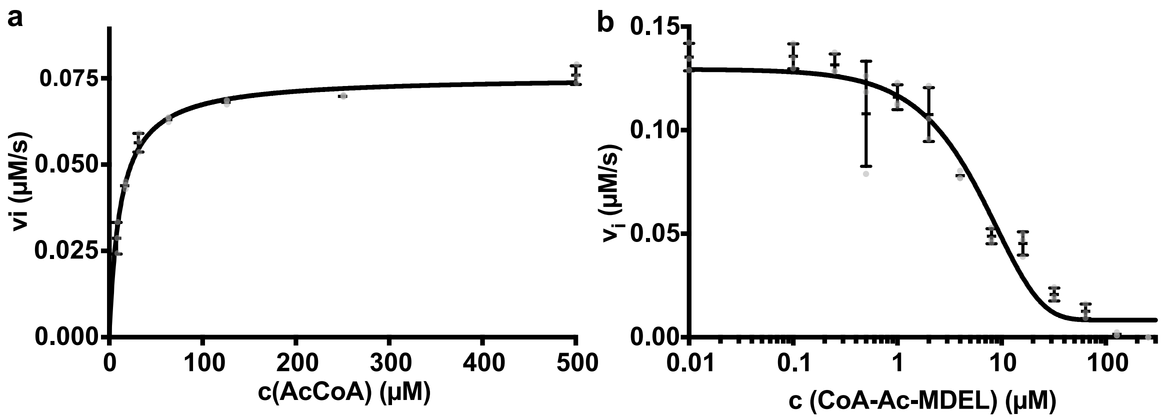

	*Ct*Naa20
K_m (µM)	12.0 ± 1.0
k_{cat} (min^{-1})	9.0 ± 0.2
k_{cat}/K_m (min^{-1} $µM^{-1}$)	0.75 ± 0.06

	*Ct*Naa20
IC_{50} (µM) CoA-Ac-MDEL	6.5 ± 2.5

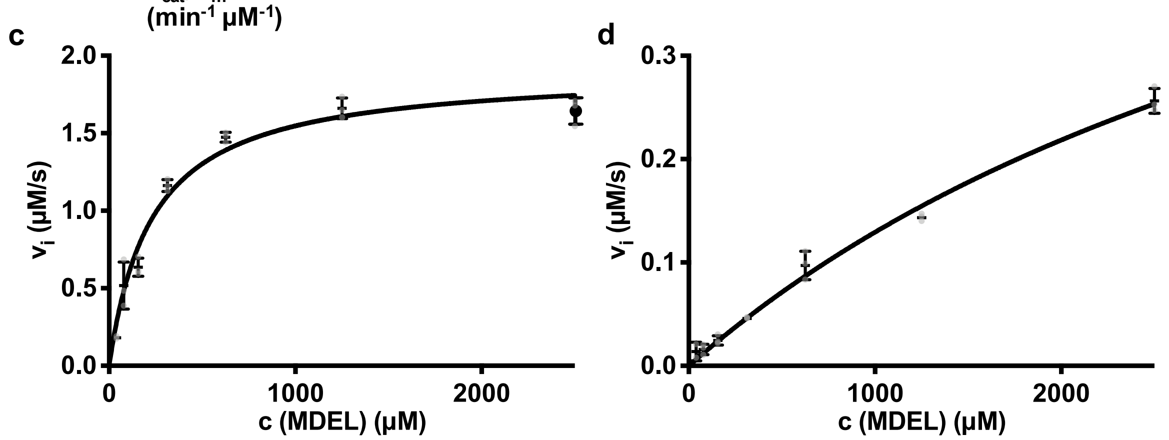

	*Ct*NatB	*Ct*Naa20
K_m (µM)	232 ± 28	4437 ± 898
k_{cat} (min^{-1})	228.7 ± 8.1	84.4 ± 12.1
k_{cat}/K_m (min^{-1} $µM^{-1}$)	0.99 ± 0.12	0.02 ± 0.01

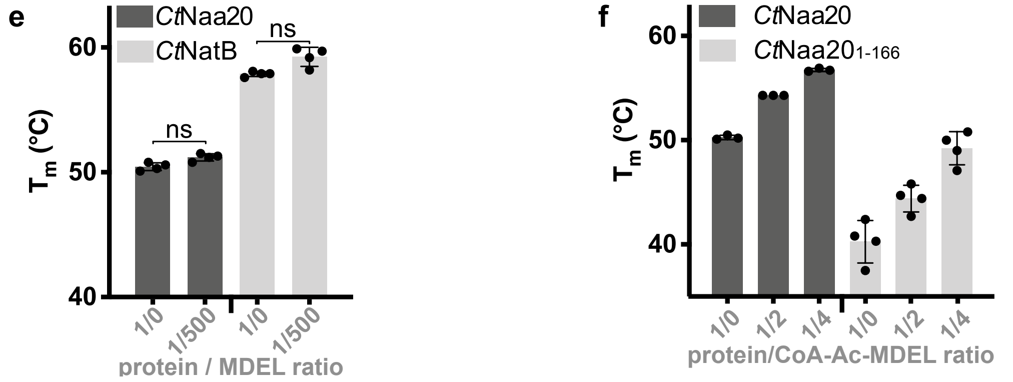

Supplementary Figure 4: *Ct*NatB and *Ct*Naa20 activity and inhibition tests. a: Michaelis-Menten plot of *Ct*Naa20 with varying AcCoA concentrations. **b:** Dose-response curve with IC_{50} value for *Ct*Naa20, inhbited by CoA-Ac-MDEL. **c:** Michaelis-Menten plot of *Ct*NatB with varying MDEL concentration. **d:** Michaelis-Menten plot of *Ct*Naa20 with varying MDEL concentration. The corresponding enzymatic parameters are shown in tables underneath the graphs. **e:** Melting temperatures of *Ct*Naa20 and *Ct*NatB in the presence of MDEL. For statistical analysis a two-sided t-test was performed: p-value(Naa20) = 0.0127; p-value(NatB) = 0.0129. ns = not significant **f:** Melting temperatures of *Ct*Naa20 and *Ct*Naa20$_{1-166}$ in the presence of CoA-Ac-MDEL. For all experiments, measurements were performed in triplicates (a-d) or quadruplicates (e-f) and error bars represent SD.

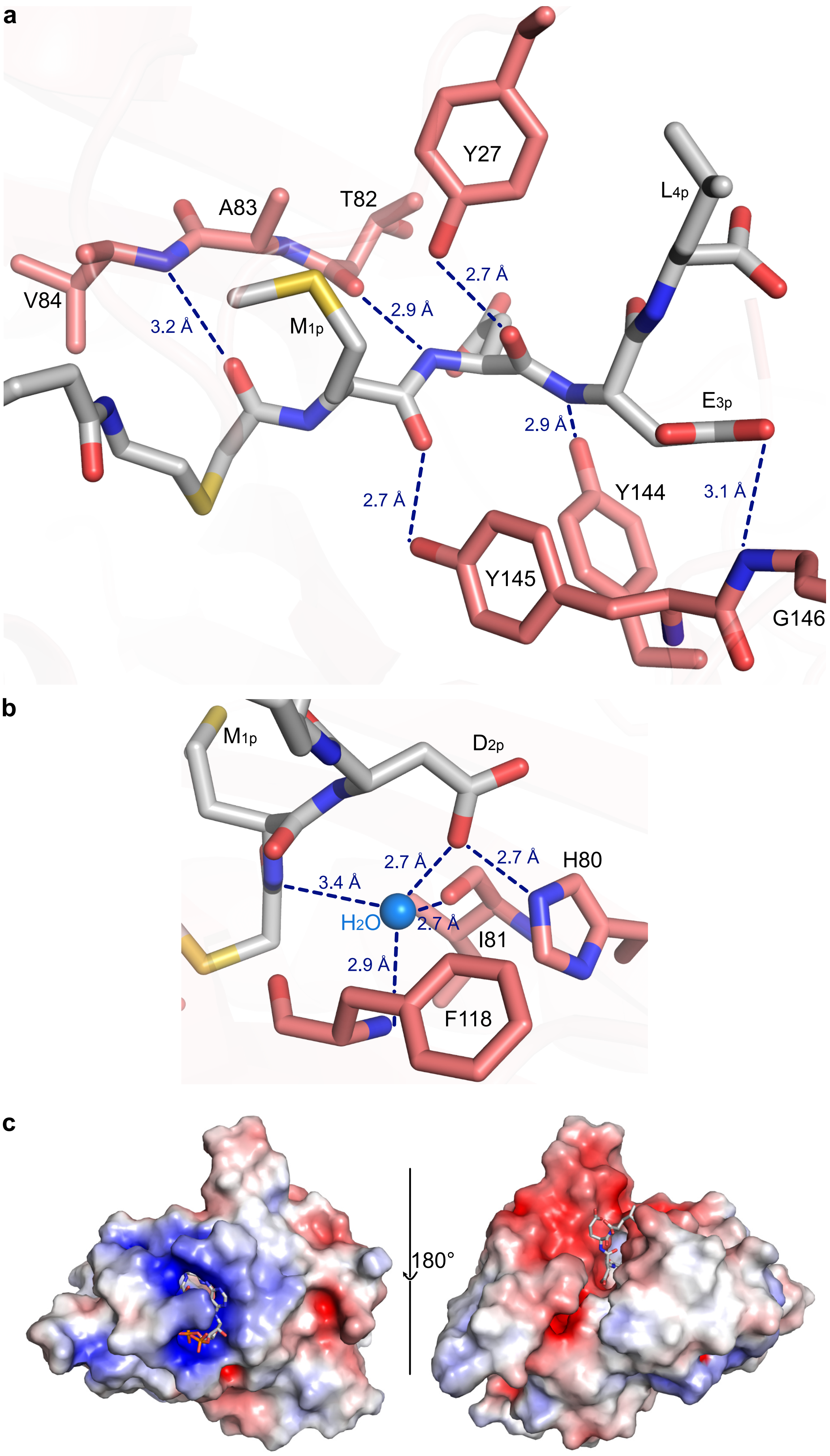
a
Y27
L4p
A83
T82
V84
M1p
3.2 Å
2.9 Å
2.7 Å
E3p
2.9 Å
2.7 Å
Y144
3.1 Å
Y145
G146
b
M1p
D2p
2.7 Å
2.7 Å
H80
3.4 Å
H2O
2.7 Å
I81
2.9 Å
F118
c
180°

Supplementary Figure 5: Protein-ligand interactions and electrostatic surface of *Ct*Naa20. a: Hydrogen bonds bewteen *Ct*Naa20 and CoA-Ac-MDEL are visualized with blue dotted lines and the corresponding atomic distances. **b:** Coordination of the active site water. The water is shown as blue sphere with the corresponding hydrogen bonds as dotted lines. **c:** The electrostatic surface of *Ct*Naa20 is represented with CoA-Ac-MDEL shown as sticks. Positive areas are shown in blue and negative areas in red.

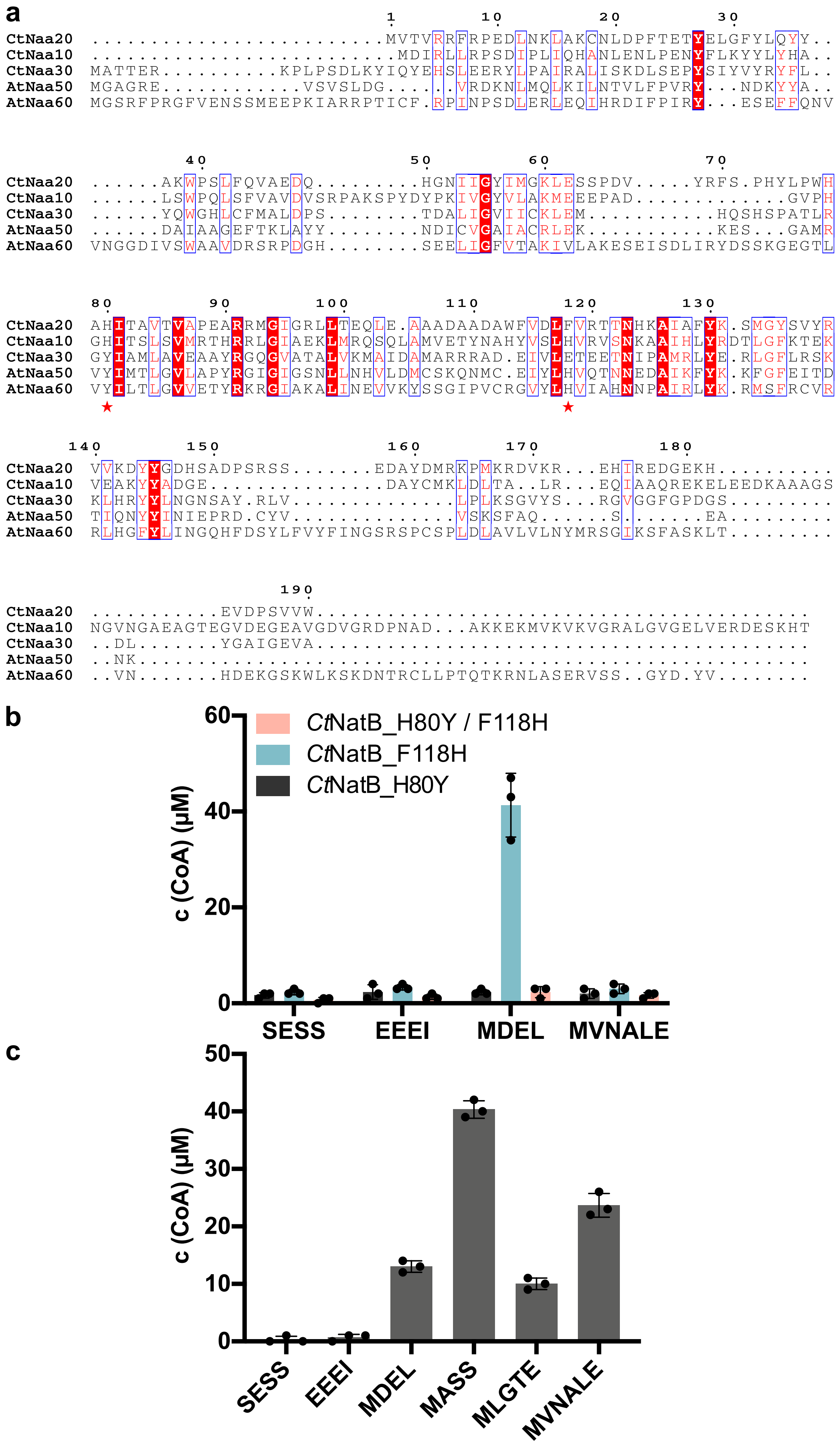
a
1 10 20 30
CtNaa20 ........................MVTVRRFRPEDLNKLAKCNLDPFTETYELGFYLQYY..
CtNaa10 ..........................MDIRLLRPSDIPLIQHANLENLPENYFLKYYLYHA..
CtNaa30 MATTER..........KPLPSDLKYIQYEHSLEERYLPAIRALISKDLSEPYSIYVYRYFL..
AtNaa50 MGAGRE............VSVSLDG.....VRDKNLMQLKILNTVLFPVRY...NDKYYA..
AtNaa60 MGSRFPRGFVENSSMEEPKIARRPTICF.RPINPSDLERLEQIHRDIFPIRY...ESEFFQNV
40 50 60 70
CtNaa20 ......AKWPSLFQVAEDQ.........HGNIIGYIMGKLESSPDV.....YRFS.PHYLPWH
CtNaa10 ......LSWPQLSFVAVDVSRPAKSPYDYPKIVGYVLAKMEEEPAD.............GVPH
CtNaa30 ......YQWGHLCFMALDPS........TDALIGVIICKLEM...........HQSHSPATLR
AtNaa50 ......DAIAAGEFTKLAYY........NDICVGAIACRLEK...........KES...GAMR
AtNaa60 VNGGDIVSWAAVDRSRPDGH........SEELIGFVTAKIVLAKESEISDLIRYDSSKGEGTL
80 90 100 110 120 130
CtNaa20 AHITAVTVAPEARRMGIGRLLTEQLE.AAADAADAWFVDLFVRTTNHKAIAFYK.SMGYSVYR
CtNaa10 GHITSLSVMRTHRRLGIAEKLMRQSQLAMVETYNAHYVSLHVRVSNKAAIHLYRDTLGFKTEK
CtNaa30 GYIAMLAVEAAYRGQGVATALVKMAIDAMARRRAD.EIVLETEETNIPAMRLYE.RLGFLRSK
AtNaa50 VYIMTLGVLAPYRGIGIGSNLLNHVLDMCSKQNMC.EIYLHVQTNNEDAIKFYK.KFGFEITD
AtNaa60 VYILTLGVVETYRKRGIAKALINEVVKYSSGIPVCRGVYLHVIAHNNPAIRLYK.RMSFRCVR
140 150 160 170 180
CtNaa20 VVKDYYGDHSADPSRSS.......EDAYDMRKPMKRDVKR...EHIREDGEKH..........
CtNaa10 VEAKYYADGE...............DAYCMKLDLTA..LR...EQIAAQREKELEEDKAAAGS
CtNaa30 KLHRYYLNGNSAY.RLV.............LPLKSGVYS...RGVGGFGPDGS.........
AtNaa50 TIQNYYINIEPRD.CYV.............VSKSFAQ.....S.......EA.........
AtNaa60 RLHGFYLINGQHFDSYLFVYFINGSRSPCSPLDLAVLVLNYMRSGIKSFASKLT........
190
CtNaa20 ....EVDPSVVW.......................................
CtNaa10 NGVNGAEAGTEGVDEGEAVGDVGRDPNAD...AKKEKMVKVKVGRALGVGELVERDESKHT
CtNaa30 ..DL.......YGAIGEVA.......................................
AtNaa50 ..NK..
AtNaa60 ..VN.......HDEKGSKWLKSKDNTRCLLPTQTKRNLASERVSS..GYD.YV........
b
CtNatB_H80Y / F118H
CtNatB_F118H
CtNatB_H80Y
c (CoA) (µM)
0
20
40
60
SESS
EEEI
MDEL
MVNALE
c
c (CoA) (µM)
0
10
20
30
40
50
SESS
EEEI
MDEL
MASS
MLGTE
MVNALE

Supplementary Figure 6: Sequence alignment of Naa20 with other NAT catalytic subunits with substrate specificity of CtNatB mutants and *At*Naa60-Y115H. a: Sequence alignment of *Ct*Naa20, *Ct*Naa10, *Ct*Naa30, *At*Naa50 and *At*Naa60. The sequence alignments were performed using Clustal Omega and ESPript3[1,2]. Mutated residues are marked with a red star *Ct: Chaetomium thermophilum; At: Arabidopsis thaliana*. **b:** Substrate specificity of the H80Y, F118H and H80Y/F118H *Ct*NatB mutants. **c:** Substrate specificity of the *At*Naa60_Y115H mutant. The *At*Naa60 wild-type does not acetylate MDEL[5]. Measurements were performed in triplicates and error bars represent the standard deviation.

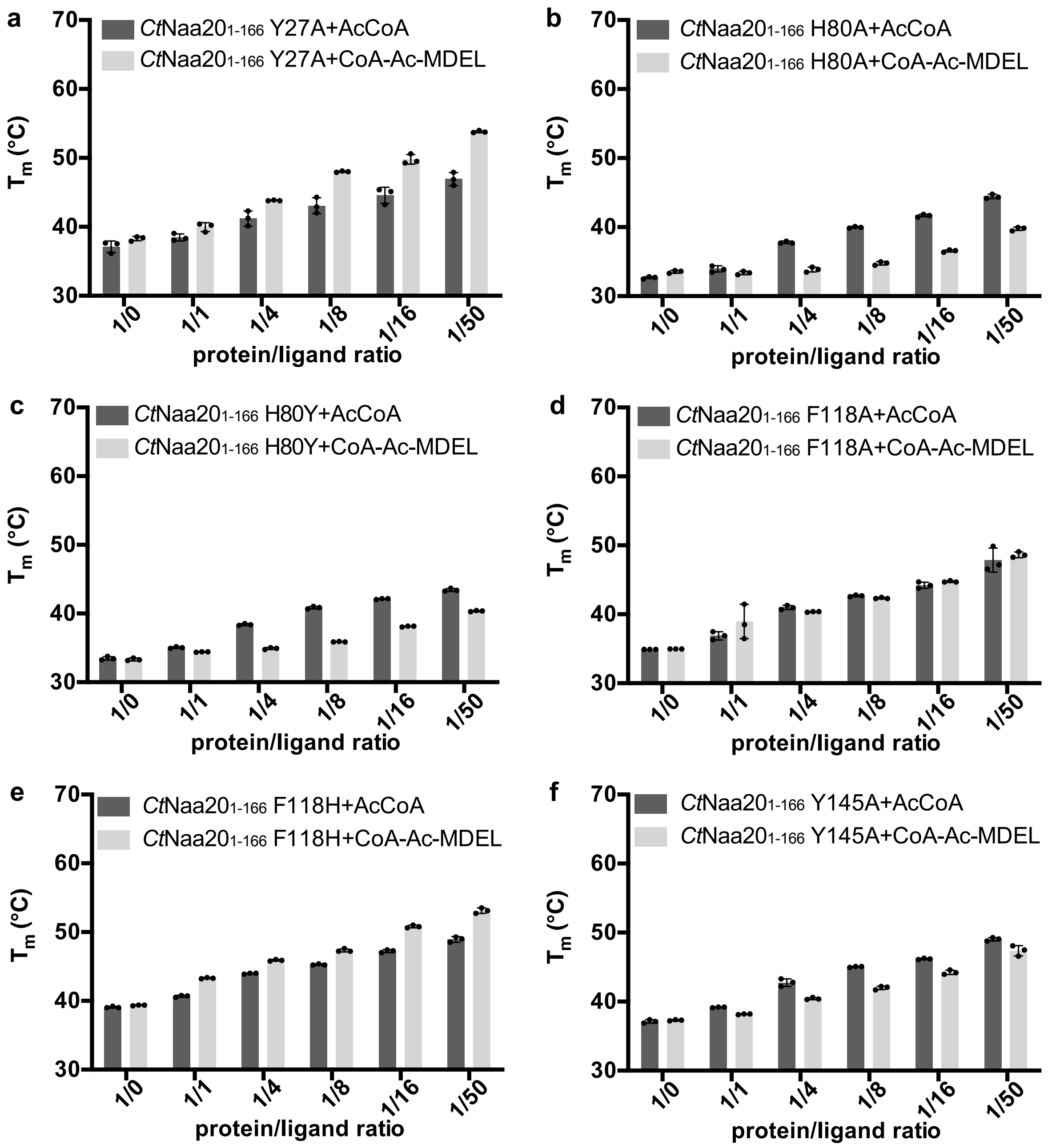

Supplementary Figure 7: Interaction of Naa20 mutants with AcCoA and CoA-Ac-MDEL. Melting temperatures of the *Ct*Naa20$_{1-166}$ mutants Y27A **(a)**, H80A (**b)**, H80Y (**c),** F118A **(d)**, F118H **(e)** and the Y145A mutant **(f)** measured via nanoDSF in the presence of varying concentrations of AcCoA or CoA-Ac-MDEL. Measurements were performed in triplicates and error bars represent the standard deviations.

Supplementary Table 1: List of primers

Primer name	Primer Sequence 5´- 3´
*Ct*Naa25_NcO_for	gcttccatgggctccatgtgttaccgttatggc
*Ct*Naa25_Ba_rev	gcttggatccctactcccacctaaccattcc
*Ct*Naa20_delNcO_for	ttctctccccactatctcccttggcatgcccatatcacagct
*Ct*Naa20_delNcO_rev	agctgtgatatgggcatgccaagggagatagtggggagagaa
*Ct*Naa20_NcO_for	gcttccatgggcgtcaccgtgcgtaggttcc
*Ct*Naa20_fl_Bam_His_rev	gcttggatccttagtgatggtgatggtgatgcaggaagtgctgctcgg
*Ct*Naa20_166_Bam_His_rev	gcttggatccttagtgatggtgatggtgatgcatcggtttacgcatatcgtac
*Ct*Naa20_N13A_for	gttgcattttgccagtttggcgagatcttctggccggaac
*Ct*Naa20_N13A_rev	gttccggccagaagatctcgccaaactggcaaaatgcaac
*Ct*Naa20_Y27A_for	ggacccgttcacggagacagctgagctgggc
*Ct*Naa20_Y27A_rev	gcccagctcagctgtctccgtgaacgggtcc
*Ct*Naa20_Y27F_for	ccgttcacggagacatttgagctgggcttct
*Ct*Naa20_Y27F_rev	agaagcccagctcaaatgtctccgtgaacgg
*Ct*Naa20_H80A_for	atctcccatggcatgccgctatcacagctgtcacag
*Ct*Naa20_H80A_rev	ctgtgacagctgtgatagcggcatgccatgggagat
*Ct*Naa20_E89A_for	cagtcgctcccgcggcccgcagaat
*Ct*Naa20_E89A_rev	attctgcgggccgcgggagcgactg
*Ct*Naa20_F118A_for	ctggttcgtcgacctggctgtgcgcactaccaac
*Ct*Naa20_F118A_rev	gttggtagtgcgcacagccaggtcgacgaaccag
*Ct*Naa20_Y145A_for	cgtgtcgttaaagactacgctggcgaccactccgc
*Ct*Naa20_Y145A_rev	gcggagtggtcgccagcgtagtctttaacgacacg
*Ct*ES27_for	gagactagaattctaatacgactcactatagggcctctagccgggcaacc ggccggcggctcgctctagag
*Ct*ES27_rev	atgtcaagcttcggcggctttcgtcctcacggactcatcaggctctatctctag agcgagccgcc

Supplementary References

1. Robert, X. & Gouet, P. Deciphering key features in protein structures with the new ENDscript server. *Nucleic acids research* **42**, W320-W324 (2014).

2. Sievers, F. & Higgins, D.G. Clustal omega. *Current protocols in bioinformatics* **48**, 3.13. 1-3.13. 16 (2014).

3. Hong, H. et al. Molecular Basis of Substrate Specific Acetylation by N-Terminal Acetyltransferase NatB. *Structure* **25**, 641-649. e3 (2017).

4. Huber, M. et al. NatB-mediated N-terminal acetylation affects growth and abiotic stress responses. *Plant physiology* **182**, 792-806 (2019).

5. Linster, E. et al. The Arabidopsis Nα-acetyltransferase NAA60 locates to the plasma membrane and is vital for the high salt stress response. *New Phytologist* **228**, 554-569 (2020).
