# Supplementary material for: Structural basis of Naa20 activity towards a canonical NatB substrate
Source: Commun Biol. 2021 Jan 4;4:2. doi: 10.1038/s42003-020-01546-4 (PMC7782713; doi:10.1038/s42003-020-01546-4)
Supplement: Supplementary file 3 — Description of Additional Supplementary Files [file 42003_2020_1546_MOESM3_ESM.pdf]

Description of Additional Supplementary Files

Supplementary Data 1: Source data underlying plots shown in figures.
